# Bone Morphogenetic Protein-8B Expression is Induced in Steatotic Hepatocytes and Promotes Hepatic Steatosis and Inflammation In Vitro

**DOI:** 10.3390/cells8050457

**Published:** 2019-05-15

**Authors:** Abdo Mahli, Tatjana Seitz, Tobias Beckröge, Kim Freese, Wolfgang Erwin Thasler, Matthias Benkert, Peter Dietrich, Ralf Weiskirchen, Anja Bosserhoff, Claus Hellerbrand

**Affiliations:** 1 Institute of Biochemistry (Emil-Fischer Zentrum), Friedrich-Alexander University Erlangen-Nürnberg, Fahrstr. 17, D-91054 Erlangen, Germany; Abdo.mahli@fau.de (A.M.); Tatjana.Seitz@fau.de (T.S.); Tobias.Beckroege@fau.de (T.B.); Kim.Freese@fau.de (K.F.); Matthias.Benkert@fau.de (M.B.); Peter.Dietrich@fau.de (P.D.); Anja.Bosserhoff@fau.de (A.B.); 2 Hepacult GmbH, Am Klopferspitz 19, D-82152 Planegg/Martinsried, Germany; Wolfgang.Thasler@swmbrk.de; 3 Institute of Molecular Pathobiochemistry, Experimental Gene Therapy and Clinical Chemistry (IFMPEGKC), RWTH University Hospital Aachen, D-52074 Aachen, Germany; rweiskirchen@ukaachen.de

**Keywords:** BMP8B, steatosis, inflammation, NAFLD, non-alcoholic fatty liver disease

## Abstract

Non-alcoholic fatty liver disease (NAFLD) is considered to be the hepatic manifestation of the metabolic syndrome. The bone morphogenetic protein-8B (BMP8B) has been shown to be expressed in brown adipose tissues and the hypothalamus and to affect thermogenesis and susceptibility to diet-induced obesity. Here, we aimed to analyze BMP8B expression in NAFLD and to gain insight into BMP8B effects on pathophysiological steps of NAFLD progression. BMP8B mRNA and protein expression were dose-dependently induced in primary human hepatocytes in vitro upon incubation with fatty acids. Furthermore, hepatic BMP8B expression was significantly increased in a murine NAFLD model and in NAFLD patients compared with controls. Incubation with recombinant BMP8B further enhanced the fatty acid-induced cellular lipid accumulation as well as NFκB activation and pro-inflammatory gene expression in hepatocytes, while siRNA-mediated BMP8B depletion ameliorated these fatty acid-induced effects. Analysis of the expression of key factors of hepatocellular lipid transport and metabolisms indicated that BMP8B effects on fatty acid uptake as well as de novo lipogenesis contributed to hepatocellular accumulation of fatty acids leading to increased storage in the form of triglycerides and enhanced combustion by beta oxidation. In conclusion, our data indicate that BMP8B enhances different pathophysiological steps of NAFLD progression and suggest BMP8B as a promising prognostic marker and therapeutic target for NAFLD and, potentially, also for other chronic liver diseases.

## 1. Introduction

Non-alcoholic fatty liver disease (NAFLD) is the leading etiology underlying chronic liver injury worldwide. It affects both adults and children and emerges as the leading cause of end-stage liver disease in the coming decades [1]. In most cases, NAFLD is associated with obesity and insulin resistance. Therefore, NAFLD is today regarded as the hepatic manifestation of the metabolic syndrome.

The spectrum of NAFLD includes a wide range of pathological conditions, from simple steatosis to non-alcoholic steatohepatitis (NASH) and cirrhosis [2].

Hepatocellular fat accumulation is the first pathological step in NAFLD. Steatosis can induce lipotoxicity, (oxidative) stress, and cellular injury. Subsequently, injured hepatocytes release different pro-inflammatory cytokines and chemokines, which can trigger hepatic inflammation. Both sustained inflammation and hepatocellular injury can induce hepatic fibrosis [3,4].

So far, no established medical therapies for the prevention or treatment of NAFLD exist. Therefore, there is a high medical need for new therapeutic targets and new forms of therapies, respectively, to beneficially affect the development and progression of NAFLD.

Bone morphogenetic proteins (BMPs) belong to the transforming growth factor-β (TGF-β) family. Currently, more than 20 different BMPs are known. They are multifunctional cytokines, which were originally discovered for their role in bone and cartilage formation and repair [5]. Furthermore, BMPs have been shown to play a critical role in early development, including organogenesis and cell differentiation. More recently, it has been discovered that BMPs are also critically involved in adult homeostasis, regulating diverse cellular processes in different organ systems. In the liver, BMPs have been mostly studied in the context of liver regeneration in response to various insults as well as in the development and progression of hepatic fibrosis in different pathological conditions (reviewed in [6]). Furthermore, BMPs have been shown to affect systemic energy balance by targeting the pancreas as well as brown and white adipose tissues (reviewed in [7]).

Recent studies have shown that bone morphogenetic protein-8B (BMP8B) is expressed in mature brown adipocytes and that BMP8B amplifies the thermogenic response in brown adipose tissue [8,9]. BMP8B-deficient mice revealed a reduced thermogenic response and an impairment in diet- and cold-induced thermogenesis [9]. Moreover, BMP8B was found to be expressed in the hypothalamus [10]. Central administration of BMP8B induced thermogenesis and increased the core temperature, leading to weight loss [10]. This effect in the hypothalamus was shown to be AMPK-dependent and to result in the sympathetic activation of the brown adipose tissue, without any change in the feeding behavior [9,10]. Furthermore, BMP8B has been shown to affect the proliferation and maturation of germ-line cells [11], while there are few studies of BMP8B expression and effects in cancer [12,13,14]. The expression and function of BMP8B in NAFLD have not been analyzed.

In this study, we analyzed the expression of BMP8B in an in vitro model of hepatic steatosis as well as in a murine NAFLD model and in liver tissues of NAFLD patients. Furthermore, we investigated the effect of BMP8B in functional in vitro models of lipid accumulation and steatosis-induced pro-inflammatory gene expression.

## 2. Materials and Methods

### 2.1. Cells and Cell Culture

Isolation and culture of primary human hepatocytes (PHH) were performed as described [15,16]. HepG2 cells (ATCC HB-8065) from ATCC (Manassas, VA, USA) were cultured as described [17].

PHH and HepG2 cells were analyzed in an in vitro model of hepatic steatosis as described [18]. Briefly, the cells were incubated with the fatty acid oleate complexed to bovine serum albumin (BSA) for 24 h. This in vitro model of hepatocellular steatosis induced by specific oleate concentrations is characterized by pathological alterations similar to those found in the hepatic tissue of NAFLD patients [18,19].

In some experimental conditions, the cells were co-incubated with recombinant BMP8B (9316-BP-025; R&D System, Minneapolis, MN, USA) in concentrations similar to those previously used in other in vitro studies [9,20]. Furthermore, the cells were stimulated with epinephrine (Sigma, Taufkirchen, Germany) in experiments analyzing the beta-adrenergic response, at a dose of 100 nM as described [21].

Human liver tissues for cell isolation or BMP8B analysis were obtained, and experimental procedures were performed according to the guidelines of the non-profit state-controlled HTCR (Human Tissue and Cell Research) foundation [22] with the informed patients’ consent.

Furthermore, BMP8B expression was analyzed in an established mouse model of NAFLD [23]. Briefly, female C57BL/6N mice (12 weeks of age) were fed either with a control diet or a Western-type diet (WTD) for 12 weeks. WTD was enriched with pork lard (15%), beef tallow (15%), palmitic acid (4%), stearic acid (4%), cholesterol (0.2%), and sucrose (30%). Both chows were prepared by Ssniff (Soest, Germany). 

### 2.2. BMP8B Depletion with Si-RNA-Pools

A total of 25 × 10^4^ cells were seeded per well in six-well plates. The Lipofectamine RNAimax transfection reagent was used (Life Technologies, Darmstadt, Germany). Si-RNA pools against the human BMP8B mRNAs were used (functionally verified by siTOOLs Biotech GmbH, Planegg, Germany). SiPool are complex pools of accurately defined siRNAs which show most efficient and robust target gene knockdown and are believed to significantly reduce off-target effects [24]. After 72 h of transfection, the functional role of BMP8B depletion was further analyzed.

### 2.3. Lipid Analysis

Lipid droplets were visualized by Oil Red O Staining as described [25]. Total triglycerides were extracted and quantified by a triglyceride determination kit (GPO) (Sigma, Deisenhofen, Germany) as described [26]. Free fatty acids in the cell culture supernatant were analyzed using a free fatty acids kit (Half-micro test) (Roche Diagnostics, Mannheim, Germany) following the manufacturer’s instructions and as described [27].

### 2.4. Quantitative Real-Time PCR Analysis

RNA isolation from cultured cells and reverse transcription were performed as described [28]. Quantitative real-time PCR was performed by applying the LightCycler technology (Roche Diagnostics, Mannheim, Germany) and using sets of specific primers as described [29]. Amplification of cDNA derived from 18S rRNA was used for normalization.

### 2.5. Protein Analysis

Protein extraction and Western blotting were performed as described [30], by applying anti-rabbit antibodies against BMP8B (#PAB25185) from Abnova (Jhouzih St., Taipei, Taiwan; diluted 1:500) and phospho-IκBα (#2859) and phospho-NFκBα p65 (#3033) antibodies from Cell Signaling Technology (Danvers, MA, USA) (all diluted 1:1000). Furthermore, antibodies against actin (sc-2955, from Santa Cruz Biotechnology, Santa Cruz, CA, USA; 1:5000) or against DGAT2 (orb19428, from Biorbyt, Cambridgeshire, UK; 1:1000) were applied. For densitometry analysis, Licor Image Studio Lite software (Licor) was used. For (immuno)histochemical analysis, standard 5 µm sections of formalin-fixed and paraffin-embedded tissue blocks were stained using the BMP8B (#PAB25185) antibody (diluted 1:50).

### 2.6. Analysis of Cell Proliferation

The colorimetric XTT assay (Roche) was used according to the manufacturer’s instructions for quantification of cell proliferation [30].

### 2.7. Statistical Analysis

Results are expressed as mean ± SEM. Comparisons between groups were performed using the unpaired Student’s t-test, one-way ANOVA, or two-way ANOVA, as appropriate. A *p* value < 0.05 was considered statistically significant. All calculations were carried out using the statistical computer package GraphPad Prism version 6.00 for Windows (GraphPad Software, San Diego, CA, USA).

## 3. Results

### 3.1. Effect of Steatosis on Hepatic BMP8B Expression

**Figure 1:**
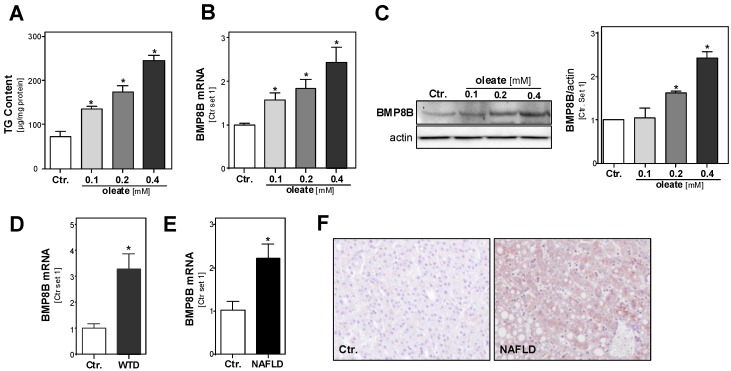
Effect of hepatic steatosis on bone morphogenetic protein-8B (BMP8B) expression. (**A**) Triglyceride (TG) content; (**B**) BMP8B mRNA expression analyzed by qRT-PCR and (**C**) Western blot (**left** panel), and densitometric analysis (**right** panel) of BMP8B protein levels in primary human hepatocytes (PHH) treated with different doses of the fatty acid oleate for 24 h. (**D**) Hepatic mRNA levels in mice were fed with non-alcoholic fatty liver disease (NAFLD)-inducing Western-type diet (WTD) and in controls (ctr.; six mice per group). (**E**) Hepatic mRNA levels of BMP8B in human NAFLD tissues (*n* = 23) compared with control liver tissues (*n* = 12). (**F**) Representative images (20×) of BMP8B immunohistochemical staining of liver tissues of NAFLD patients and controls; (*: *p* < 0.05, using one way ANOVA (**A**–**C**) and Student’s t-test (**D**,**E**)).

To assess the effect of hepatocellular lipid accumulation on BMP8B expression, we applied an established in vitro model of hepatic steatosis [18]. Incubation of PHH with serial concentrations of the fatty acid oleate (complexed to albumin) led to a dose-dependent increase of cellular triglyceride levels within 24 h (Figure 1A). We have previously shown that hepatocellular steatosis induced by these oleate concentrations leads to pathological alterations similar to those found in hepatic tissue of NAFLD patients [18,19]. Interestingly, we found that the induction of lipid accumulation also dose-dependently induced the expression of BMP8B mRNA (Figure 1B) and protein (Figure 1C) in PHH. Next, we analyzed BMP8B expression in an established murine NAFLD model; we have previously shown that mice fed with a steatosis-inducing WTD showed similar pathological changes as those found in NAFLD patients [23]. BMP8B expression levels were significantly higher in the livers of WTD-fed mice compared with control mice (Figure 1D). Similarly, we observed significantly higher BMP8B mRNA expression levels in human NAFLD tissues compared with non-steatotic liver tissue from control patients (Figure 1E). Immunohistochemical analysis confirmed BMP8B expression in steatotic hepatocytes in human NAFLD tissues, while no immune signal was observed in control liver tissue (Figure 1F). Together, these data indicate that steatosis induces BMP8B expression in hepatocytes.

### 3.2. Effect of the BMP8B on Steatosis in Hepatocytes

**Figure 2:**
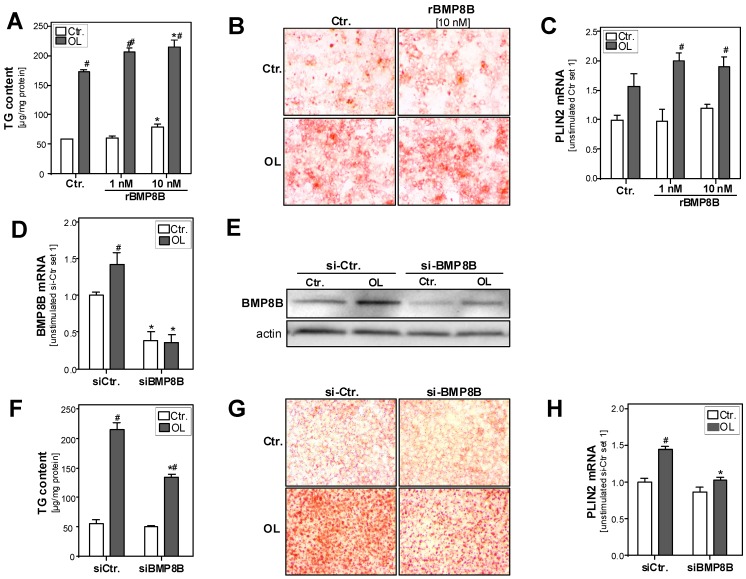
Effect of BMP8B (depletion) on hepatocellular steatosis in vitro. (**A**–**C**): PHH were incubated with oleate (OL; 0.4 mM) for 24 h in the presence or absence of recombinant BMP8B (rBMP8B, 1 nM or 10 nM). (**A**) Hepatocellular TG content normalized to protein content; (**B**) Microscopic images (20×) of Oil red O staining; (**C**) PLIN2 mRNA levels analyzed by quantitative RT-PCR, (* *p* < 0.05 compared with the corresponding condition without BMP8B stimulation; # *p* < 0.05 compared with the corresponding condition without oleate stimulation using two-way ANOVA). (**D**–**H**): BMP8B mRNA expression was depleted in HepG2 hepatoma cells by transfection with si-RNA (si-BMP8B); control cells were transfected with control siRNA (si-Ctr). Subsequently, the cells were incubated with or without 0.4 mM oleate for additional 24 h. (**D**) Hepatocellular BMP8B mRNA levels; (**E**) Western blot analysis of BMP8B protein levels; (**F**) Hepatocellular TG content; (**G**) Microscopic images (20×) of Oil red O staining; (**H**) Hepatocellular PLIN2 mRNA levels analyzed by quantitative RT-PCR; * *p* < 0.05 compared with si-Ctr-transfected cells; # *p* < 0.05 compared with cells without oleate stimulation; two-way ANOVA).

Next, we wanted to investigate whether BMP8B also affects the hepatocellular lipid accumulation and metabolism by applying the above described in vitro model of hepatic steatosis. In this model, PHH were incubated with 1 nM or 10 nM recombinant BMP8B (rBMP8B). We applied similar rBMP8B doses as in previously published studies assessing BMP8B effects on the lipolytic activity in differentiated brown adipocytes or on vascular tube formation in cardiac endothelial cells [9,20]. Incubation with rBMP8B for 24 h slightly increased triglycerides content in control PHH (Figure 2A). Furthermore, already the low rBMP8B dose (1 nM) significantly enhanced fatty acid-induced triglyceride accumulation in hepatocytes (Figure 2A). Oil red O staining confirmed the significantly enhanced accumulation of lipid droplets in rBMP8B-treated PHH (Figure 2B). Fitting to this, the expression levels of the lipid droplet protein perilipin-2 (PLIN2) were significantly higher in rBMP8B-treated cells compared with control cells (Figure 2C).

In a complementary approach, we analyzed the effect of BMP8B suppression on basal as well as fatty acid-induced steatosis in the human hepatoma cell line HepG2. Transfection with si-RNA significantly reduced basal as well as fatty acid-induced BMP8B expression compared with control si-RNA-transfected cells (Figure 2D,E). Assessment of cellular triglycerides revealed significantly reduced levels in BMP8B-depleted cells compared with control cells after fatty acid incubation (Figure 2F). Oil red O staining intensity was reduced in BMP8B-depleted cells (Figure 2G). Furthermore, PLIN2 expression levels were significantly lower in BMP8B-depleted cells (Figure 2H).

In summary, these findings show that exogenous (recombinant) BMP8B as well as endogenous BMP8B expression promote fatty acid induced-hepatocellular steatosis.

### 3.3. Effect of the BMP8B on Lipid Metabolism in Hepatocytes

**Figure 3:**
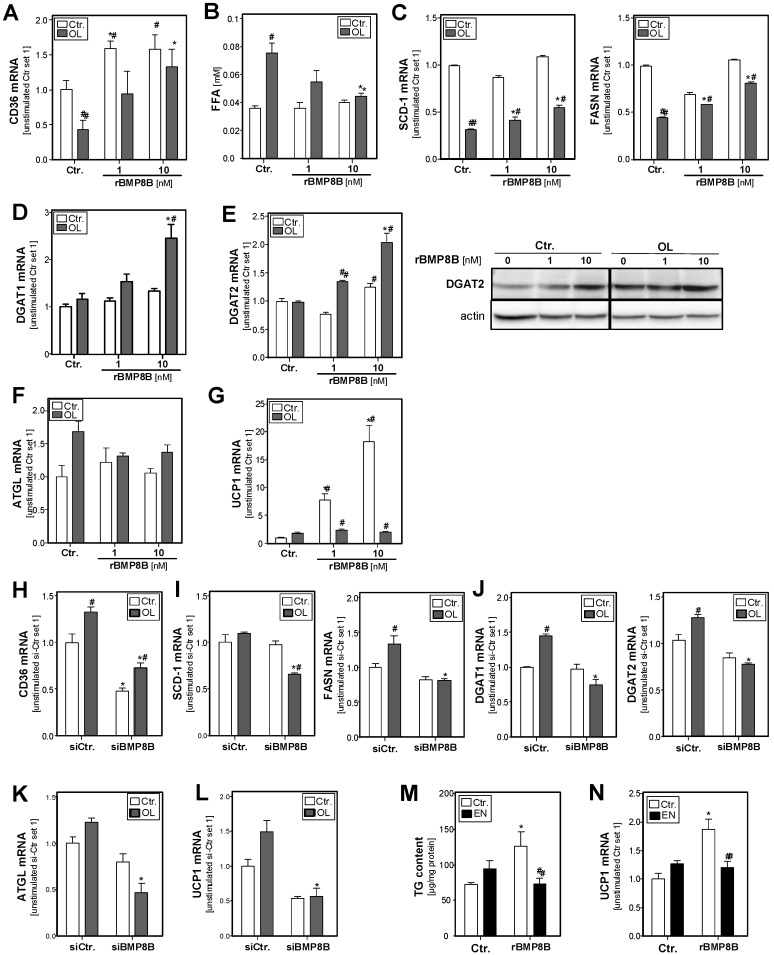
Effect of BMP8B (depletion) on hepatocellular lipid metabolism. (**A**–**G**): PHH were incubated with OL (0.4 mM) for 24 h in the presence or absence of recombinant rBMP8B. (**H**–**L**): BMP8B mRNA expression was depleted in HepG2 hepatoma cells by transfection with si-RNA (si-BMP8B); control cells were transfected with control-siRNA (si-Ctr). Subsequently, the cells were incubated with or without 0.4 mM OL for additional 24 h. (**M**–**N**): HepG2 cells were incubated with rBMP8B (10 nM) for 24 h in the presence or absence of epinephrine (EN; 100 nM). mRNA levels of (**A**,**H**) CD36, (**C**,**I**) SCD1 and FASN, (**D**, **E** and **J**, **left** panel) DGAT1 and DGAT2, (**F**,**K**) ATGL, and (**G**,**L**) UCP1 analyzed by quantitative RT-PCR; (**B**) Free fatty acids levels in cell culture supernatant; (**E**, **right** panel) Western blot analysis of DGAT2 protein levels; (**M**) Cellular TG content normalized to protein content; (**N**) mRNA levels of UCP1. (**A**–**G**): * *p* < 0.05 compared with the corresponding condition without BMP8B stimulation; # *p* < 0.05 compared with the corresponding condition without OL stimulation; (**H**–**L**): * *p* < 0.05 compared with si-Ctr-transfected cells; # *p* < 0.05 compared with cells without OL stimulation. (**M**–**N**): * *p* < 0.05 compared with cells without rBMP8B stimulation; # *p* < 0.05 compared with cells without epinephrine stimulation; two-way ANOVA).

In search for the underlying mechanisms of BMP8B effects on hepatocellular steatosis, we analyzed BMP8B effects on the expression of key factors of hepatocellular lipid transport and metabolisms, which are frequently altered in NAFLD patients (reviewed in [31]). CD36 is a critical transporter for fatty acid uptake in hepatocytes [32]. Interestingly, treatment of PHH with rBMP8B caused a dose-dependent induction of CD36 expression (Figure 3A). In line with this, analysis of fatty acid content in the supernatant revealed lower levels in BMP8B-treated hepatocytes, indicative of enhanced fatty acid uptake (Figure 3B). Fatty acid synthase (FASN) and stearoyl-CoA desaturase 1 (SCD-1) are central regulators of de novo lipogenesis. The expression of both enzymes was reduced in response to fatty acid-induced lipid accumulation, and this downregulation was in part abrogated by rBMP8B (Figure 3C). In line with the increase of cellular fatty acid levels, the expression of diacylglycerol *O*-acyltransferase 1 and 2 (DGAT-1 and DGAT-2), which catalyze the final reaction in the synthesis of triglycerides, was induced in BMP8B-treated cells (Figure 3D,E). 

Adipose triglyceride lipase (ATGL) plays a critical role in hepatic lipid turnover, and it has been shown that BMP8B promotes lipolysis in adipocyte [9]. However, in our model system, we did not observe significant effects of BMP8B on ATGL expression in control or lipid-loaded hepatocytes **(**Figure 3F). 

Furthermore, BMP8B has been reported to promote thermogenesis in brown adipocytes [9]. Interestingly, we observed a significant and dose-dependent upregulation of the expression of uncoupling protein 1 (UCP1) in hepatocytes in response to treatment with BMP8B (Figure 3G). Increased UCP1 expression could point to the uncoupling of mitochondria and dissipation of biochemical energy.

Conversely, BMP8B-depleted HepG2 cells showed reduced CD36 expression in control conditions as well as upon fatty acid treatment (Figure 3H). Upon fatty acid treatment, SCD-1 and FASN mRNA levels were lower in BMP8B-depleted cells (Figure 3I). Furthermore, basal as well as fatty acid-induced expression levels of DGAT-1 and DGAT-2 were reduced in BMP8B-depleted cells (Figure 3J). Surprisingly, also ATGL expression levels were slightly reduced in BMP8b-depleted cells (Figure 3K). Moreover, and complementary to the increased UCP1 expression in BMP8b-treated cells, UCP1 mRNA levels were lower in BMP8B-depleted cells compared with control cells (Figure 3L). 

One further previously shown biological function of BMP8B is its effect on the adrenergic-induced remodeling of the neuro-vascular network in adipose tissue [20]. Therefore, we also wanted to analyze the impact of BMP8B on the β-adrenergic response in hepatocytes. Stimulation with epinephrine caused only a slight induction of cellular triglyceride levels (Figure 3M). Interestingly, combined application of BMP8B and epinephrine equalized the steatosis-inducing effects of the single compounds (Figure 3M). Furthermore, we assessed the effect of epinephrine on UCP1 expression in hepatocytes; here, we observed only a slightly induced UCP1 expression in our model system (Figure 3N). However, epinephrine treatment abrogated BMP8B-induced upregulation of UCP1 expression (Figure 3N).

Together, these data indicate that BMP8B effects on fatty acid uptake as well as de novo lipogenesis contribute to hepatocellular accumulation of fatty acids, leading to increased storage in the form of triglycerides. However, and interestingly, our data also give an indication that BMP8B can affect the beta-adrenergic response and the mitochondria membrane potential in a way counteracting steatosis. Still, in total, the pro-steatotic effect of BMP8B appeared to dominate in hepatocytes.

### 3.4. Effect of the BMP8B on Steatosis-Induced Pro-Inflammatory Gene Expression in Hepatocytes

**Figure 4:**
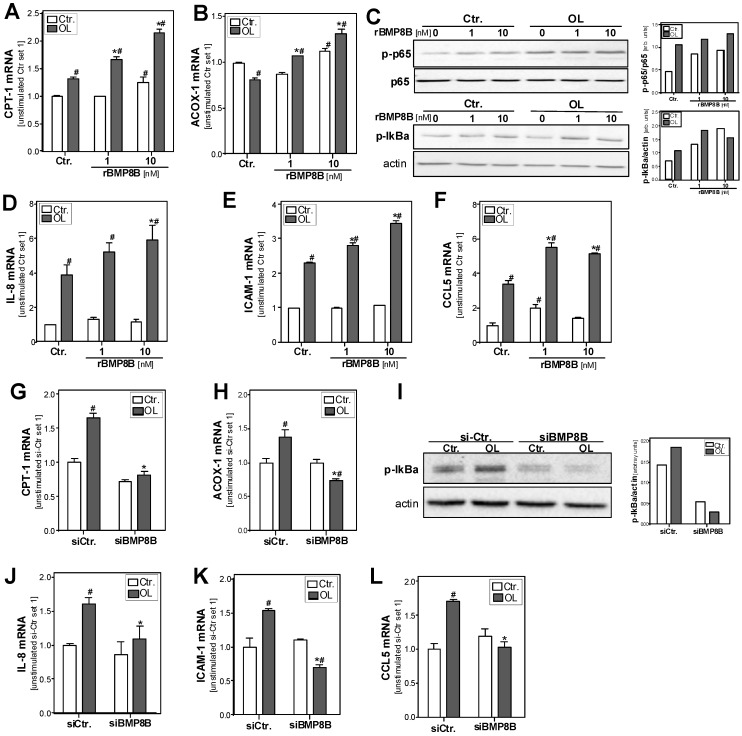
Effect of BMP8B (depletion) on hepatocellular oxidative stress and inflammation. (**A**–**F**): PHH were incubated with the fatty acid OL (0.4 mM) for 24 h in the presence or absence of recombinant rBMP8B. (**G**–**L**): BMP8B mRNA expression was depleted in HepG2 hepatoma cells by transfection with si-RNA (si-BMP8B); control cells were transfected with control-siRNA (si-Ctr). Subsequently, the cells were incubated with or without 0.4 mM OL for additional 24 h. Cellular mRNA levels of CPT-1 (**A**,**G**) and ACOX-1 (**B**,**H**) analyzed by quantitative RT-PCR. (**C**,**I**) Western blot analysis of p-p65 and p-IκBα protein levels; unphosphorylated p65 and actin served as control for loading adjustment. The right panels depict the densitometric analysis. Cellular mRNA levels of (**D**,**J**) IL-8, (**E**,**K**) ICAM-1, and (**F**,**L**) CCL5 analyzed by quantitative RT-PCR. (**A**–**F**): * *p* < 0.05 compared with the corresponding condition without BMP8B stimulation; # *p* < 0.05 compared with the corresponding condition without OL stimulation; (**G**–**L**): * *p* < 0.05 compared with si-Ctr-transfected cells; # *p* < 0.05 compared with cells without OL stimulation; two-way ANOVA).

Hepatocellular lipid accumulation induces fatty acid combustion. In line with this, BMP8B treatment dose-dependently induced the expression of carnitine palmitoyltransferase I (CPT1), a key enzyme of mitochondrial β-oxidation (Figure 4A). Incubation with the high BMP8B dose (10 nM) also induced the expression of acyl-CoA oxidase 1 (ACOX1), a key enzyme of peroxisomal β-oxidation (Figure 4B).

Beta-oxidation of fatty acids causes the formation of reactive oxygen species, which can cause oxidative stress, a well-known promoter of inflammation in hepatocytes and a critical factor for the progression from simple steatosis to NASH [33]. Furthermore, steatosis is a known inducer of endoplasmic reticulum (ER) stress in hepatocytes [34].

ER stress as well as oxidative stress can cause the activation of the transcription factor nuclear factor-kappa-B (NFκB) [35], and NFκB activation is known to play a critical role in the pathogenesis of NAFLD [36]. In its inactive form, NFκB is bound to its inhibitor IκBα in the cytoplasm. NFκB-inducing stimuli lead to the phosphorylation and subsequent degradation of IκBα. As a consequence, the p65 subunit of the NFκB complex gets phosphorylated, translocates into the nucleus, and becomes transcriptionally active. Western blot analysis revealed higher phospo-IκBα and phospo-p65 levels in fatty acid-treated hepatocytes, indicating NFκB activation (Figure 4C). Noteworthy, BMP8B treatment further and dose-dependently increased both basal and fatty acid-induced levels of phospo-IκBα and phospo-p65 (Figure 4C). Interleukin-8 (IL-8) and intercellular adhesion molecule 1 (ICAM-1) are known NFκB targets and are both pro-inflammatory molecules also known to promote hepatic inflammation in NAFLD [37,38]. Also the expression of the chemokine (C-C motif) ligand 5 (CCL5) is regulated by NFκB activity and has been correlated with inflammation and disease progression in NAFLD [39,40,41]. Interestingly, BMP8B treatment caused a dose-dependent induction of IL-8, ICAM-1, and CCL5 in steatotic hepatocytes (Figure 4D–F).

On the contrary, CPT-1 and ACOX-1 expression were reduced in fatty acid-treated and BMP8B-depleted HepG2 cells compared with controls (Figure 4G,H). Furthermore, BMP8B depletion resulted in lower levels of phosphorylated IκBα (Figure 4I). Moreover, the fatty acid-induced expression of IL-8, ICAM-1, and CCL5 was ameliorated in BMP8B-depleted cells (Figure 4J–L).

These findings indicate that BMP8B promotes steatosis-induced pro-inflammatory gene expression in hepatocytes. Moreover, it appears that BMP8B also induces (pro-inflammatory) NFκB activity in control hepatocytes.

## 4. Discussion

NAFLD is today recognized as the most frequent liver disease worldwide, and, together with the increase of obesity, its incidence is also expected to further increase [42]. There is a close connection between NAFLD and the metabolic syndrome, and in regard to NAFLD prevention and therapy, it is important to note that improvement of individual components of the metabolic syndrome (e.g., body weight reduction, improvement of diabetes, or correction of dyslipidemia) also have a beneficial effect on NAFLD [43].

BMP8B is expressed in mature brown adipocytes and amplifies the thermogenic response in the brown adipose tissue [9,20]. Furthermore, BMP8B is expressed in the hypothalamus, where it induces thermogenesis and increases the core temperature, leading to weight loss [10]. Conversely, BMP8B-deficient mice exhibit altered brown adipose tissue with impaired thermogenesis and susceptibility to diet-induced obesity [9]. In this study, we identified hepatocytes as a further source of BMP8B and found that BMP8B expression is further upregulated by lipid accumulation in hepatocytes in vitro as well as in the liver of mice fed with steatosis-inducing Western-type diet and in hepatic tissues of NAFLD patients. We found that BMP8B expression further increased in patients with NASH as compared with patients with simple steatosis (data not shown). This suggests that, in addition to steatosis, further mechanisms may induce hepatic BMP8B expression, which needs to be evaluated in future studies.

In the present study, we furthermore assessed the impact of BMP8B on hepatocellular lipid accumulation and metabolism in an in vitro model of hepatocellular steatosis [18]. Here, BMP8B depletion reduced fatty acid-induced steatosis, while exogenous BMP8B induced lipid accumulation in hepatocytes. Analysis of gene expression of key enzymes of hepatocellular lipid accumulation indicated that BMP8B promoted the uptake of fatty acids into hepatocytes. Furthermore, inhibitory effects of BMP8B on the steatosis-induced downregulation of key enzymes of de novo lipogenesis seem to contribute to its pro-steatotic effect in hepatocytes.

Previous studies revealed that BMP8B promotes lipolysis in adipocytes [9]. In the present study, we did not find a significant effect of BMP8B on the expression of ATGL, a critical enzyme in hepatic lipid turnover, indicating that BMP8B does not affect lipolysis in hepatocytes. However, and interestingly, we found that BMP8B significantly induced the expression of UCP1 in hepatocytes. It has been reported that BMP8B promotes thermogenesis in brown adipocytes [9], and our data indicate that BMP8B might also causes uncoupling of mitochondria in hepatocytes. Furthermore, it is interesting that the combined application of BMP8B and epinephrine equalized the steatosis-inducing effects of the single compounds in our in vitro model of hepatocellular steatosis. These data suggest that BMP8B might exhibit anti-steatotic as well as pro-steatotic effects on hepatocytes. Still, the steatosis-inducing effect of BMP8B is dominant in hepatocytes in vitro. Hepatic steatosis is also associated with ER and oxidative cellular stress, which can induce the activation of the transcription factor NFκB [35]. In line with this, we observed that BMP8B further enhanced fatty acid-induced NFκB activation and pro-inflammatory gene expression in hepatocytes. Together, these data indicate BMP8B as a promoter of hepatocellular steatosis and inflammation. Still, one may speculate that, depending on the stage of the disease or in defined situations such as enhanced beta-adrenergic activity, BMP8B may also exhibit anti-steatotic effects. Future studies including analyses in preclinical mouse models are necessary to fully elucidate the role of BMP8B during the development and progression of NAFLD.

On the basis of previous studies describing BMP8B expression in brown adipose tissue and the hypothalamus as a critical regulator of thermogenesis and a modulator of obesity, the focus of the present study was on NAFLD and fatty acid-induced alterations in hepatocytes. Still, it has to be noted that BMP8B depletion or stimulation with recombinant BMP8B, respectively, also affected basal NFκB activity and pro-inflammatory gene expression in hepatocytes. This suggests that BMP8B also plays a pathophysiological role in other (chronic) liver diseases, which also needs to be addressed in future studies. Furthermore, BMP8B has been shown to promote the progression of pancreatic cancer [12], while conflicting studies exist regarding its role in gastric cancer [13,14]. Future studies will need to investigate the expression and function of BMP8B in the development and progression of hepatocellular carcinoma.

In summary, the present study has newly revealed increased hepatocellular BMP8B expression in liver steatosis and the impact of BMP8B on hepatic steatosis and inflammation, which are critical pathophysiological steps of NAFLD. Our data indicate the potential of BMP8B as a novel prognostic marker and therapeutic target for NAFLD and, possibly, also for other forms of chronic liver disease.
